# Microbiota Reconstitution Does Not Cause Bone Loss in Germ-Free Mice

**DOI:** 10.1128/mSphereDirect.00545-17

**Published:** 2018-01-03

**Authors:** Darin Quach, Fraser Collins, Narayanan Parameswaran, Laura McCabe, Robert A. Britton

**Affiliations:** a Department of Microbiology and Molecular Genetics, Michigan State University, East Lansing, Michigan, USA; b Department of Physiology, Michigan State University, East Lansing, Michigan, USA; c Department of Molecular Virology and Microbiology, Alkek Center for Metagenomics and Microbiome Research, Baylor College of Medicine, Houston, Texas, USA; University of Iowa; Indiana University School of Medicine; Georgia State University

**Keywords:** bone, microbiome, microbiota, osteoporosis

## Abstract

Annually, an estimated 2 million osteoporotic fractures occur in the United States alone. Osteoporosis imparts a great burden on the health care system. The identification of novel regulators of bone health is critical for developing more effective therapeutics. A previous study on the colonization of germ-free (GF) mice with a microbial community has demonstrated that bacterial colonization dramatically increases bone loss. We therefore investigated the impact of multiple microbial communities in different mice to understand how generalizable the impact of bacterial colonization is on bone health. To investigate the impact of different microbial communities on bone health in outbred and inbred mouse strains, gavage was performed on GF Swiss Webster and GF C57BL/6 mice to introduce distinct microbiotas that originated from either humans or mice. GF mice displayed a high degree of colonization, as indicated by more than 90% of the operational taxonomic units present in the starting inoculum being successfully colonized in the mice when they were examined at the end of the experiment. In spite of the successful colonization of GF mice with gut microbiota of either mouse or human origin, bone mass did not change significantly in any of the groups tested. Furthermore, static and dynamic bone parameters and osteoclast precursor and T cell populations, as well as the expression of several inflammatory markers, were mostly unchanged following microbial colonization of GF mice.

**IMPORTANCE** The microbiota has been shown to be an important regulator of health and development. With regard to its effect on bone health, a previous study has suggested that gut microbes negatively impact bone density. However, we show here that this is not generalizable to all microbial communities and mouse strain backgrounds. Our results demonstrate that colonization of mice, both outbred and inbred strains, did not have a major impact on bone health. The identification of microbial communities that do not negatively impact bone health may provide a foundation for future investigations that seek to identify microbes that are either beneficial or detrimental to bone metabolism.

## Abstract (precis)

The microbiota has been shown to be an important regulator of health and development. With regard to its effect on bone health, a previous study has suggested that gut microbes negatively impact bone density. However, we show here that this is not generalizable to all microbial communities and mouse strain backgrounds. Our results demonstrate that colonization of mice, both outbred and inbred strains, did not have a major impact on bone health. The identification of microbial communities that do not negatively impact bone health may provide a foundation for future investigations that seek to identify microbes that are either beneficial or detrimental to bone metabolism.

## INTRODUCTION

The intestinal microbiota is the collection of microbes, including bacteria, archaea, viruses, helminths, and fungi, that inhabit our gut. At birth, humans become exposed to microorganisms that establish colonization at various body sites, including the skin, mouth, gastrointestinal (GI) tract, and vaginal epithelium. These body sites serve as the interface that fosters interactions between the microbes and their respective host. Furthermore, several studies have demonstrated the importance of the microbiota in the development and maintenance of health. For example, germ-free (GF) mice have decreased gut lymphoid tissue development, as well as altered microvillus architecture (1, 2). Moreover, GF mice have decreased expression levels of pattern recognition receptors that detect microbes (e.g., Toll and NOD-like), as well as of antimicrobial peptides (e.g., Reg3γ), which strongly highlights how the microbiota impacts the functional role of the immune system (2, 3). In animal disease models, the ability to mount an immune response is also hindered in GF animals and restored upon conventionalization (i.e., restoration of microbiota from an animal raised under standard laboratory conditions) (4, 5).

Recently, more emphasis has been placed on the role of the microbiota in skeletal health (6, 7). The rationale for this is based on the known impact of the microbiota on multiple facets of the immune system, the importance of the immune system in bone remodeling, and the gut that serves as a messenger between the two (8–10). Bone remodeling, which is composed of the coupled processes of bone formation and bone resorption, undergoes various phases throughout life. In early human life, bone formation outweighs bone resorption and contributes to increased bone deposition until a plateau is reached in early adulthood (11). Then, a shift in favor of bone resorption takes place, with bone loss gradually increasing over time. Different pathologies can accelerate bone loss by impacting the processes of bone formation by osteoblasts and bone resorption by osteoclasts (OCL). Under many different pathological bone conditions, the primary driver of bone loss is increased osteoclastic bone resorption that is oftentimes mediated by immune signaling (12, 13).

Osteoclasts originate from monocytic precursors in the bone marrow, and many studies have demonstrated their interaction with and regulation by immune cells like B and T cells (14–17). In addition to impacting the local immune response in the gut, the microbiota has also been shown to regulate the immune response and hematopoiesis at distant sites, including the bone marrow (18). Thus, we and others hypothesized that the gut microbiota is an important factor that impacts bone health through its regulation of the immune system (7, 19, 20).

A previous study has shown that bone density is negatively regulated by the presence of the microbiota. By comparing female GF mice and conventionalized (CONV-D) mice in the C57BL/6 genetic background, it was shown that the gut microbiota markedly decreased bone mass (19). In addition, CONV-D mice also displayed an expansion in osteoclast precursor cells, CD4^+^ T cells, and serum serotonin levels. These results were intriguing given that increased immune cell populations and serotonin levels are important driving factors in the development of osteoporosis (8, 21, 22). In direct contrast, Schwarzer et al. demonstrated that various bone parameters associated with bone health were decreased in GF BALB/c animals compared to the results for wild-type (WT) mice (23). More recently, estrogen deficiency-induced bone loss was shown to require the presence of the microbiota in order for bone loss to occur (20).

Although these studies indicated that bone density is regulated by the presence of the microbiota, the conflicting results did not resolve the roles that specific microbial community member(s) play in determining bone health. Therefore, in this study, we set out to identify specific bacterial species that impact bone health by colonizing GF Swiss Webster (SW) mice with microbial communities from diverse fecal donors and characterizing the impact of these communities on bone health. Contrary to previous results, our study demonstrates that the colonization of GF mice with several different microbial communities does not result in increased or decreased bone mass. Additionally, these studies were performed in both outbred (SW) and inbred (C57BL/6) mouse genetic backgrounds. Through comparative 16S rRNA gene analysis, we identified separate and distinct clustering of microbial communities based upon mouse genetic background. Thus, our results underscore the importance of testing different microbial communities before generalizing findings to encompass all microbiota.

(Part of this work was published in Darin Quach’s Ph.D. thesis at Michigan State University [24].)

## RESULTS

### Presence of a human gut microbiota does not impact bone mass in Swiss Webster mice.

**FIG 1:**
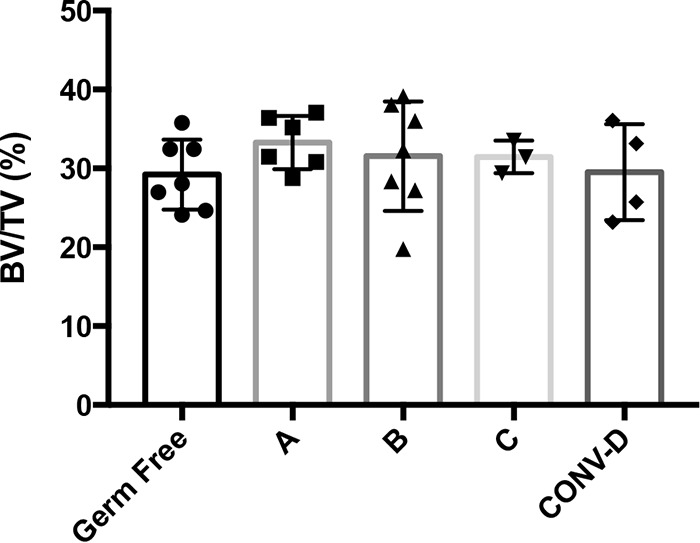
Trabecular bone volume fraction (BVF) values (shown as bone volume per total volume analyzed [BV/TV]) for GF Swiss Webster (SW) mice after colonization with different human microbiotas. Four-week-old female germ-free (GF) Swiss Webster mice were colonized with different human microbiotas (samples A to C) for 8 weeks. The GF and conventionalized (CONV-D) groups served as controls. The results for individual mice and the mean values ± SEM (*n* = 3 to 7) are shown.

To identify specific microbes that affect bone health, we investigated the impact of different human microbial communities in GF mice. Four-week-old female GF SW mice were humanized by intragastric gavage with fecal microbial communities from healthy human volunteers. In total, 3 different human microbial communities were used for colonization, as well as cecal contents from WT SW mice (Table 1). After 12 weeks of colonization, bone mass in the distal femur was measured, and we found that there was no significant difference between the bone volume fraction (BVF) values of GF mice and mice colonized with our collection of human microbiotas or the cecal content of the mouse (Fig. 1) (*P* = 0.61).

**TABLE 1:** Description of microbiotas used in mouse studies

Microbiota source	Description
CONV-D	Cecal contents of mice conventionally raised with mouse microbiota
Sample A	Fecal sample collected from healthy human vegetarian
Sample B	Sample collected at year 1 from individual who provided sample C
Sample C	Sample collected at year 0 from healthy human omnivore

Femur lengths and body weights were not significantly different among the treatment groups (Table 2). Other femur bone parameters, such as bone mineral density (BMD), trabecular thickness (Tb. Th.), trabecular number (Tb. N.), and trabecular spacing (Tb. Sp.), were similar between GF and CONV-D mice (Table 2). Cortical (Ct.) bone parameters, such as volume, thickness, and moment of inertia (MOI), were not significantly different between GF mice and mice colonized with a microbiota (Table S1 in the supplemental material). These data show that colonization of GF mice with human or mouse microbial communities had no impact on bone health in the SW outbred genetic background.

10.1128/mSphereDirect.00545-17.3 TABLE S1 Cortical bone parameters for moment of inertia (MOI) measurements in Swiss Webster mouse studies. Download TABLE S1, PDF file, 0.04 MB. Copyright © 2018 Quach et al. 2018 Quach et al. This content is distributed under the terms of the Creative Commons Attribution 4.0 International license.

**TABLE 2:** Trabecular and cortical bone parameters for humanized Swiss Webster mice^a^

Bone type, parameter	Mean value ± SEM for samples from mice receiving indicated microbiota (no. of mice)					*P* value
	GF (7)	A (6)	B (7)	C (3)	CONV-D (4)	
Trabecular						
BV/TV (%)	30.84 ± 2.40	32.80 ± 1.94	30.93 ± 3.58	31.45 ± 1.51	29.50 ± 4.50	0.88
BMD (mg/cm^3^)	225.82 ± 30.04	246.25 ± 25.52	228.57 ± 53.03	234.66 ± 20.42	220.01 ± 41.30	0.83
Tb. Th. (μm)	5.74 ± 0.71	6.44 ± 0.25	5.87 ± 1.20	6.11 ± 0.75	5.79 ± 0.56	0.64
Tb. N. (mm^−1^)	5.32 ± 0.72	5.03 ± 0.44	5.18 ± 1.01	5.12 ± 0.56	5.03 ± 1.10	0.98
Tb. sp. (μm)	133.49 ± 24.01	135.50 ± 19.41	139.58 ± 35.80	135.90 ± 15.14	148.18 ± 45.04	0.96
Cortical						
Ct. volume (mm^3^)	0.98 ± 0.01	0.98 ± 0.01	0.98 ± 0.01	0.97 ± 0.01	0.97 ± 0.01	0.08
Ct. thickness (μm)	285.57 ± 17.64	303.20 ± 20.46	301.67 ± 6.80	276.00 ± 12.77	277.50 ± 16.28	0.19
BMD (mg/cm^3^)	1,039.43 ± 8.02	1,015.99 ± 1.43	1,041.60 ± 12.61	1,041.50 ± 4.46	1,042.04 ± 18.93	0.97
MOI (mm^4^)	0.11 ± 0.03	0.09 ± 0.01	0.10 ± 0.01	0.08 ± 0.04	0.09 ± 0.01	0.30
Body weight (g)	35.04 ± 2.40	32.01 ± 1.17	33.45 ± 0.96	32.75 ± 1.15	31.74 ± 1.09	0.78
Femur length (mm)	17.22 ± 0.24	17.35 ± 0.26	17.21 ± 0.13	17.09 ± 0.14	17.10 ± 0.21	0.82

a This table summarizes data from the experiment described in the legend to Fig. 1.

### Presence of a normal mouse gut microbiota does not impact bone mass in female Swiss Webster or C57BL/6 mice.

**FIG 2:**
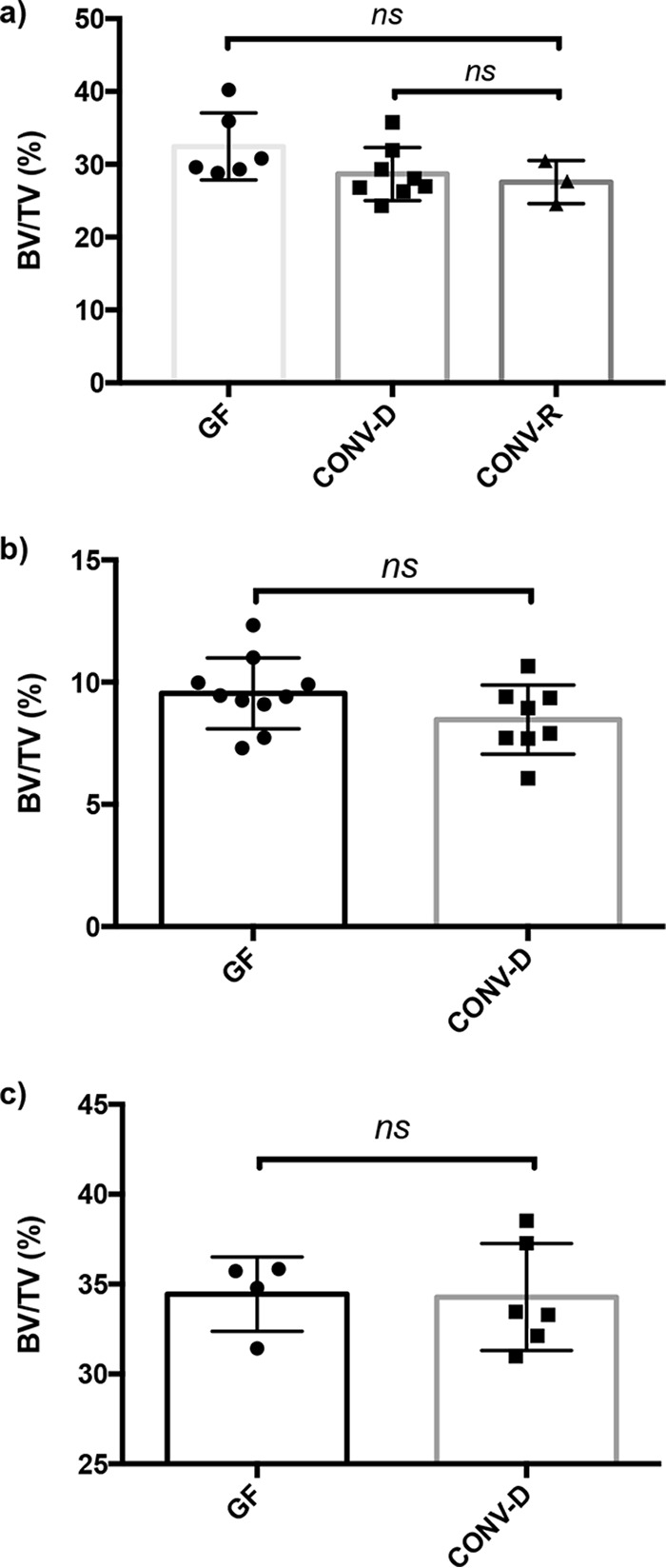
Conventionalization of female GF SW and C57BL/6 mice does not impact bone density. Female GF Swiss Webster (a) and C57BL/6 (b) mice and male GF Swiss Webster (c) mice were colonized with conventional mouse microbiota for 4 weeks (conventionalized [CONV-D]). The GF group served as the control. No differences in BVF were observed when conventionalized mice were compared to their GF counterparts. CONV-R, conventionally reared. The results for individual mice and mean values ± SEM (*n* = 3 to 10) are shown.

Previous work demonstrating a negative impact on bone mass following the reconstitution of microbiota in GF mice was performed in female C57BL/6 mice (19). To test whether the lack of impact on bone health following microbiota colonization in this study was due to the mouse genetic background, we conventionalized 4-week-old female SW and C57BL/6 GF mice with microbial communities prepared from cecal contents of genetic background-, age-, and sex-matched mice raised in the Baylor College of Medicine mouse facility. Following 4 weeks of colonization, we compared the BVFs of the distal femurs between GF and CONV-D mice and observed that there was no significant difference between the two groups in either the SW (Fig. 2a) (*P* = 0.15) or the C57BL/6 (Fig. 2b) (*P* = 0.14) genetic background. Conventionally reared (CONV-R) mice, used as a control, displayed a mean BVF that was not statistically different from that of the GF or CONV-D group (Fig. 2a).

Measurements for BMD, Tb. Th., Tb. N., and Tb. Sp. were similar between GF and CONV-D mice at 8 weeks of age in the SW background (Table 3). Statistically significant differences in Tb. N. and Tb. Sp. were observed in C57BL/6 mice despite not observing a significant difference in BVF or BMD. In addition, the lack of a significant difference in the MOI suggests that there is no difference in the bone structural strength between GF and CONV-D mice (Table S2). A comparison of the cortical volume and thickness from the midshaft of the femur also demonstrated no difference between the two groups in the C57BL/6 background (Table 4).

10.1128/mSphereDirect.00545-17.4 TABLE S2 Cortical bone parameters for MOI measurements in C57BL/6 mouse studies. Download TABLE S2, PDF file, 0.04 MB. Copyright © 2018 Quach et al. 2018 Quach et al. This content is distributed under the terms of the Creative Commons Attribution 4.0 International license.

**TABLE 3:** Trabecular and cortical bone parameters for conventionalized SW mice^a^

Bone type, parameter	Mean value ± SEM for samples from mice receiving indicated treatment (no. of mice)		*P* value
	GF (6)	CONV-D (8)	
Trabecular			
BV/TV (%)	32.16 ± 4.78	28.57 ± 3.71	0.14
BMD (mg/cm^3^)	269.73 ± 32.74	253.17 ± 28.75	0.33
Tb. Th. (μm)	56.31 ± 5.79	53.85 ± 5.21	0.42
Tb. N. (mm^−1^)	5.71 ± 0.21	5.31 ± 0.16	0.15
Tb. sp. (μm)	149.85 ± 5.77	160.55 ± 6.51	0.26
Cortical			
Ct. volume (mm^3^)	0.94 ± 0.01	0.95 ± 0.01	0.48
Ct. thickness (μm)	244.38 ± 2.63	242.15 ± 2.18	0.32
BMD (mg/cm^3^)	1,074.78 ± 9.83	1,083.66 ± 12.49	0.92
MOI (mm^4^)	0.08 ± 0.02	0.09 ± 0.02	0.84
Body weight (g)	32.82 ± 2.79	31.98 ± 2.60	0.57
Femur length (mm)	16.79 ± 0.27	16.47 ± 0.56	0.22

a This table summarizes data from the experiment described in the legend to Fig. 2a.

**TABLE 4:** Trabecular and cortical bone parameters for conventionalized C57BL/6 mice^a^

Bone type, parameter	Mean value ± SEM for samples from mice receiving indicated treatment (no. of mice)		*P* value
	GF (10)	CONV-D (8)	
Trabecular			
BV/TV (%)	9.33 ± 0.54	8.46 ± 0.49	0.26
BMD (mg/cm^3^)	109.91 ± 10.79	98.48 ± 12.09	0.07
Tb. Th. (μm)	33.45 ± 1.09	35.01 ± 2.25	0.07
Tb. N. (mm^−1^)	2.83 ± 0.40	2.41 ± 0.28	0.02
Tb. sp. (μm)	203.91 ± 14.08	229.94 ± 11.88	0.001
Cortical			
Ct. volume (mm^3^)	0.55 ± 0.03	0.56 ± 0.01	0.38
Ct. thickness (μm)	162.38 ± 2.63	166.88 ± 2.18	0.21
BMD (mg/cm^3^)	963.58 ± 23.71	954.22 ± 23.05	0.41
MOI (mm^4^)	0.030 ± 0.002	0.028 ± 0.004	0.30
Body weight (g)	18.68 ± 0.71	17.83 ± 1.43	0.12
Femur length (mm)	12.41 ± 0.62	12.29 ± 0.46	0.67

a This table summarizes data from the experiment described in the legend to Fig. 2b.

### Presence of a normal mouse gut microbiota does not impact bone mass in male Swiss Webster mice.

Sex differences have been shown to impact various facets of bone metabolism (25–27). Previous studies on the role of the gut microbiota in bone density examined female mice (19). To begin understanding whether sex plays a role in determining the impact of the microbiota on bone health, we conventionalized 4-week-old male GF Swiss Webster mice as well. Similar to the results for their female counterparts, the presence of a microbiota did not impact the BVF (Fig. 2c) (*P* = 0.9253). These results demonstrate that neither sex nor genetic background impacts bone mass in response to microbial colonization of GF mice.

### Histomorphometric analysis of the distal femur.

Histomorphometry was performed on the distal femur to determine static and dynamic bone parameters. We observed no significant differences between the number of osteoclasts (OCL) per trabecular bone surface between GF and CONV-D Swiss Webster mice. Consistent with the bone results thus far, no significant differences were observed when we compared the results in different groups for mineralizing surface per trabecular bone surface (MS/BS), bone formation rate (BFR), and mineral apposition rate (MAR) (Table 5; Fig. S1).

10.1128/mSphereDirect.00545-17.1 FIG S1 Conventionalization does not impact MAR or TRAP staining of metaphyseal region of femur. Images are representative of multiple images taken for each specimen of each animal group (*n* = 8). (a) Calcein incorporation illustrates the amount of mineral apposition over a span of 5 days. (b) TRAP staining of metaphyseal region, where osteoclasts stain purple. Download FIG S1, PDF file, 1.3 MB. Copyright © 2018 Quach et al. 2018 Quach et al. This content is distributed under the terms of the Creative Commons Attribution 4.0 International license.

**TABLE 5:** Histomorphometry of trabecular bone in distal femurs of conventionalized SW mice

Parameter	Mean value ± SEM for samples from mice receiving indicated treatment (no. of mice)		*P* value
	GF (5)	CONV-D (5)	
OCL/bone surface (%)	12.91 ± 4.01	16.41 ± 5.51	0.25
MS/BS (%)	11.41 ± 1.23	10.02 ± 0.94	0.40
BFR (μm/day)	0.19 ± 0.03	0.11 ± 0.01	0.21
MAR (μm^3^/μm^2^/day)	1.41 ± 0.54	1.4 ± 0.39	0.31

### Conventionalization does not impact T cell populations or OCL precursor cells and outgrowth in GF and CONV-D mice.

An imbalance in bone remodeling that favors osteoclastic bone resorption often leads to pathological bone loss; T-cell-driven osteoclastogenesis is one of the reasons for this osteoclastic bone resorption (22, 28). Previously, it was shown that the gut microbiota can decrease bone mass, which correlated with increases in CD4^+^ and CD8^+^ T cell populations, as well as osteoclast precursor cells (19). Therefore, we investigated whether the gut microbiota impacts bone marrow T-cell populations and/or the amount of osteoclast precursors or osteoclastogenesis in SW mice in the presence or absence of a microbiota.

**FIG 3:**
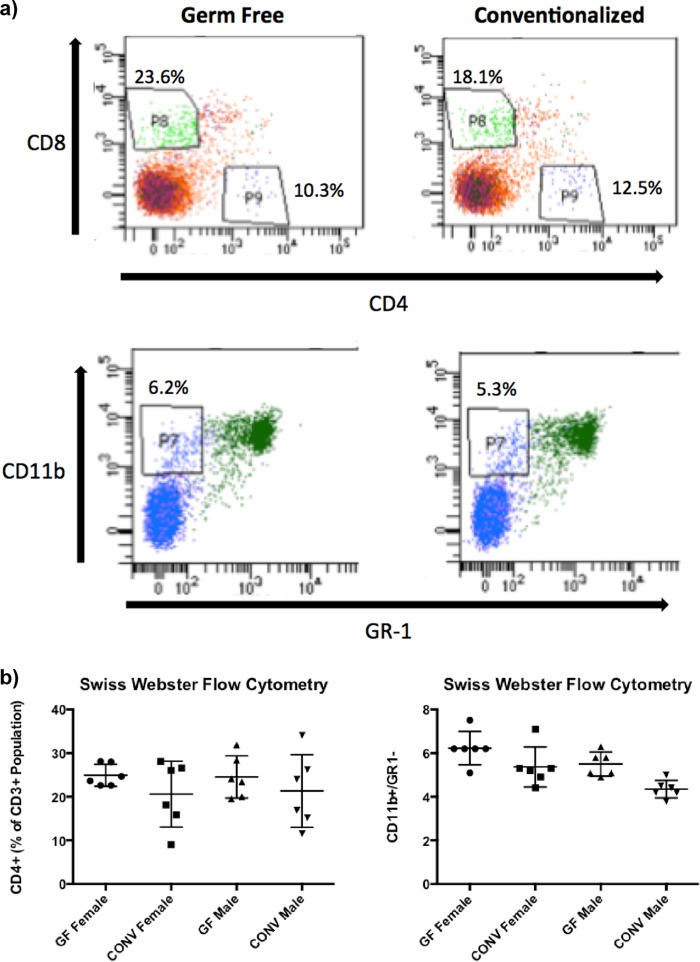
Conventionalization of GF SW mice resulted in no changes in bone marrow cell populations. Bone marrow cells were flushed from 8-week-old mice and stained for antibodies to quantify T cell populations (CD3, CD4, and CD8) and osteoclast precursors (CD11b^+^/GR1^−^) by flow cytometry. (a) Representative images of flow cytometry plots of cell percentages of total CD3^+^ population for T cells and total bone marrow population for osteoclast precursors. (b) Quantitation of the different cell populations. The results for individual mice and mean values ± SEM (*n* = 3 to 10) are shown.

**FIG 4:**
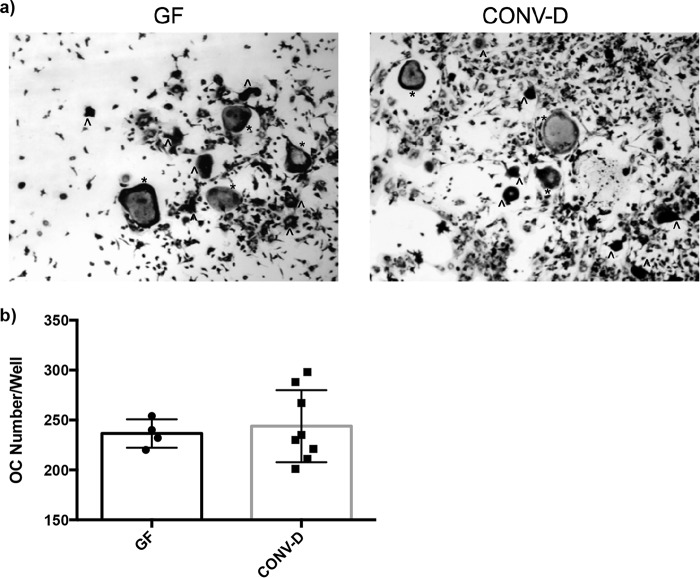
Osteoclast outgrowth from bone marrow cells of GF SW and conventionalized (CONV-D) mice. Bone marrow cells were flushed from the femurs of 8-week-old GF and CONV-D mice and stimulated for differentiation with RANKL and M-CSF. TRAP^+^ cells were quantitated after 4 days of culture. (a) Representative images of bone marrow cells from mice treated under GF and CONV-D conditions. (b) Total osteoclast (OC) numbers per well were quantitated. The results for individual mice and mean values ± SEM are shown.

Flow cytometric analyses indicated that conventionalization of GF mice in the SW genetic background did not significantly impact the bone marrow T cell populations (Fig. 3). Osteoclast differentiation was measured by quantitating the amount of giant multinucleated cells that stained positive for tartrate-resistant alkaline phosphatase (TRAP) following stimulation with receptor activator of NF-kappa B ligand (RANKL) and macrophage colony-stimulating factor (M-CSF) for 4 days. Consistent with there being no changes in the T cell and OCL precursor populations, no significant difference in OCL outgrowth was detected in conventionalized mice compared to that in their GF counterparts in both SW (Fig. 4a and b) and C57BL/6 mice (Fig. S2).

10.1128/mSphereDirect.00545-17.2 FIG S2 Colonization of female GF C57BL/6 mice does not impact osteoclast outgrowth from bone marrow cells. Female GF C57BL/6 mice were conventionalized at 4 weeks of age. Bone marrow cells were flushed from the femurs of 8-week-old GF and CONV-D mice and stimulated for differentiation with RANKL and M-CSF. TRAP^+^ cells were quantitated after 4 days of culture. Total osteoclast numbers in each well were quantitated. Data are provided as mean values ± SEM. Download FIG S2, PDF file, 0.02 MB. Copyright © 2018 Quach et al. 2018 Quach et al. This content is distributed under the terms of the Creative Commons Attribution 4.0 International license.

### Analysis of inflammatory markers.

**FIG 5:**
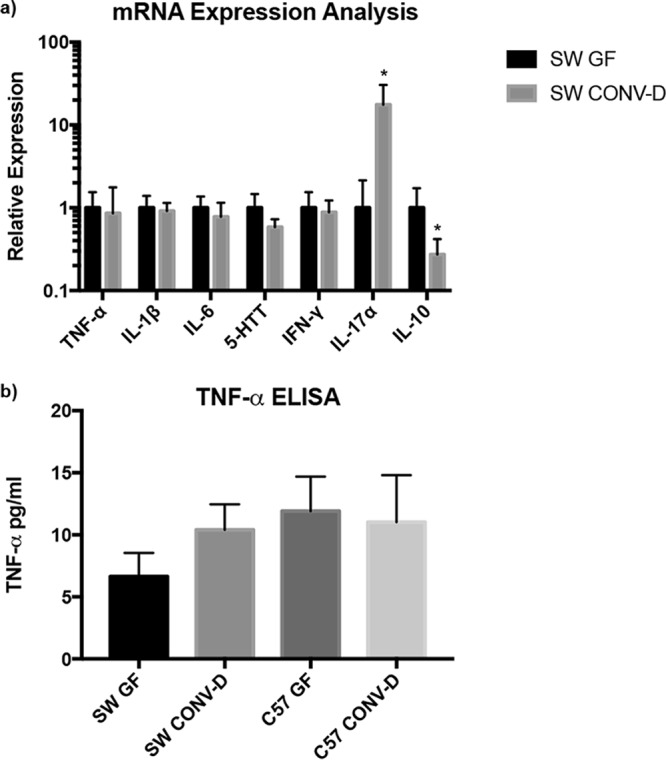
Analysis of mRNA expression in the colon and TNF-α levels in serum. (a) The colonic mRNA expression level of IL-17α was upregulated and that of IL-10 was downregulated due to conventionalization. (b) Serum levels of TNF-α were unaffected following conventionalization. ELISA, enzyme-linked immunosorbent assay. *, *P* < 0.05 with respect to the results for SW GF mice, using Student’s *t* test.

Increased levels of various inflammatory cytokines, most notably tumor necrosis factor alpha (TNF-α), have been shown to promote osteoclastogenesis and bone loss in human and animal studies (29–31). To investigate whether the production of certain cytokines was regulated by the presence of a gut microbiota, we compared the expression levels between GF and conventionalized mice by performing quantitative real-time PCR (qRT-PCR) on the TNF-α, interleukin-1β (IL-1β), IL-6, serotonin reuptake transporter (SERT), gamma interferon (IFN-γ), IL-17A, and IL-10 genes from colonic mRNA. With the exception of IL-17A mRNA expression being increased and IL-10 being decreased, no genes that were examined were differentially regulated by the reconstituted gut microbiota in GF mice (Fig. 5a). In accordance with these results, TNF-α levels in the serum were not affected by conventionalization (Fig. 5b).

### Fecal microbial community analysis.

**FIG 6:**
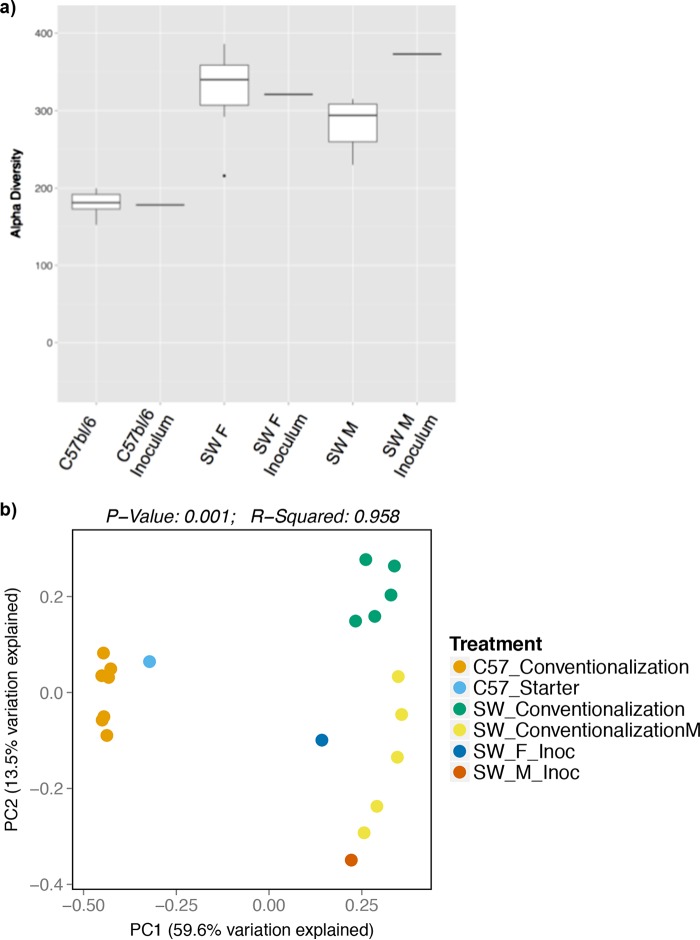
Alpha and beta diversity measures for conventionalized SW and C57BL/6 mice indicate higher diversity in SW mice and distinct clustering. (a) The comparison of OTUs in starting inocula and conventionalized mice suggests a high level of colonization and efficient microbiota transfer in both SW (male and female) and C57BL/6 mouse genetic background. (b) In a PCoA plot, the results for the conventionalized mice cluster together according to mouse genetic background and starting inocula.

For the mouse colonization studies, we analyzed the microbial communities present in the fecal samples of mice at the conclusion of the experiments by analysis of the V4 hypervariable region of the 16S rRNA gene. Alpha and beta diversity measures reflected highly efficient microbiota transfer, since most of the species identified in the inocula were present in the recipient CONV-D SW and C57BL/6 mice. Conventionalization of female C57BL/6 and SW mice identified the presence of 178 (out of 182) and 296 (out of 315), respectively, of the operational taxonomic units (OTUs) present in the original inocula (Fig. 6; Tables S3 and S4). Interestingly, we found that transfers resulted in mouse strain-specific conventionalization. Specifically, conventionalized mice of the C57BL/6 and SW genetic backgrounds shared 154 OTUs, but an additional 31 OTUs were unique to C57BL/6 mice and 168 OTUs were unique to SW mice (Tables S3 and S4).

10.1128/mSphereDirect.00545-17.5 TABLE S3 Abundances of OTUs for each mouse group. Values are reported as means within that group and as percentages of the total community. Download TABLE S3, XLSX file, 0.02 MB. Copyright © 2018 Quach et al. 2018 Quach et al. This content is distributed under the terms of the Creative Commons Attribution 4.0 International license.

10.1128/mSphereDirect.00545-17.6 TABLE S4 OTU taxonomy table. Download TABLE S4, XLSX file, 0.02 MB. Copyright © 2018 Quach et al. 2018 Quach et al. This content is distributed under the terms of the Creative Commons Attribution 4.0 International license.

## DISCUSSION

In this study, we focused on the impact of the microbiota on bone health, with the goal of identifying microbes with therapeutic potential. Previous work demonstrated that conventionalizing female GF C57BL/6 mice with a normal mouse gut microbiota decreased bone mass and negatively impacted bone health (19). Additionally, it was demonstrated by Sjögren et al. (19) that several immune and bone parameters were impacted in the presence of the microbiota and that these correlated with bone loss. Overall, our findings demonstrate that the introduction of defined microbiotas to GF animals does not significantly alter bone mass in males or females in two different mouse genetic backgrounds. Despite the fact that the expression levels of IL-10 and IL-17α in the colon (Fig. 5b) were impacted in a way that has been shown to favor the development of osteoporosis (32–34), serum levels of TNF-α and T cell populations in the bone marrow compartment were not impacted by the presence of a microbiota (Fig. 3). This highlights the need to consider the mouse background and microbial communities to be tested when considering the impact of bacterial colonization on bone health.

While there was a downward trend in bone mass in CONV-D mice (Fig. 2), the changes were minor and not significant. Only a less than 5% decrease in BVF was observed, which is in stark contrast to the 30 to 40% decrease that was previously demonstrated (19). Similarly, no significant differences in cortical volume, cortical thickness, inner and outer diameter of the cortical bone, and MOI were observed following the humanization or conventionalization of mice in comparison to the results under the GF condition (Tables 3 and 4; Tables S1 and S2).

When comparing our results with published studies, there were some factors that could account for the differences observed. First, the mode of transplantation of the microbiota differed. Both studies conventionalized their mice with cecal contents. However, as reported by Sjögren et al. (19), their conventionalization process was by coprophagy, where cecal contents were put onto the fur of the mice, whereas we conventionalized our animals by intragastric gavage after the cecal contents were prepared and maintained under anaerobic conditions. While it is hard to speculate on whether this ultimately culminated in pronounced differences in community colonization, it cannot be excluded. Another possible reason for the observed differences is the inherent differences between the microbiotas used to conventionalize mice manifesting in different bone responses. It has been well documented that mice purchased from different animal vendors and housed at different mouse facilities foster microbial community colonization that is location specific (35). Our laboratory and others have demonstrated that altering microbiome composition, through methods such as treating mice with pathogenic bacteria (36) or probiotics (20, 37, 38), can decrease or increase bone density, respectively. Since there were no published community analysis results reported by Sjögren et al. (19), it is impossible to compare their study with ours with respect to the microbial communities used for the conventionalization study.

It is worth noting that recently published studies observed different impacts on bone health following colonization of GF animals (20, 23). Specifically, one study demonstrated that conventionalization of GF mice results in an increase in several parameters of bone health (23). Under this condition, Schwarzer et al. (23) show that the presence of the microbiota was associated with increases in bone volume per total volume analyzed (BV/TV) and cortical thickness compared to those of the GF mice. In contrast, another study, in female GF mice, did not find that conventionalization affected BV/TV measures, although significant changes were observed in trabecular architecture (20). These studies further support the notion that bone health is highly regulated by the functional composition of the microbiota. The current compositional analysis of the microbial analysis only provides information about what is present or was once present. Even if the microbial compositions of two communities are different, the functions of these communities may not differ. This is discussed further in a review by Yan and Charles (39). To gain insight into functionalities being carried out by a specific community, future studies focused on identifying specific microbial members impacting bone health, as well as integrating metagenomics, transcriptomics, and proteomics, may be necessary.

### Conclusions.

The impact of intestinal microbes on bone metabolism has received much attention in the last 5 years, with the initial publication in this area suggesting a negative impact of gut bacteria on bone health. We have systematically tested several different microbial communities in SW and C57BL/6 mice and have observed little impact on bone metabolism. The results demonstrate that a negative effect of bacteria on bone health cannot be generalized.

## MATERIALS AND METHODS

### GF mouse husbandry.

The Institutional Animal Care and Use Committee (IACUC) at Baylor College of Medicine approved all the protocols (protocol no. AN-6676) performed in this study. Germ-free (GF) wild-type (WT) mice in the SW and C57BL/6 genetic background were generated by the Baylor College of Medicine GF mouse facility. Germ-free mice were housed in flexible-film isolators (Class Biologically Clean, Madison, WI) supplied with HEPA-filtered air. The room’s light cycle was 12 h light/12 h dark. The mice were given autoclaved food (5V5R; LabDiet, St. Louis, MO) and sterile tap water. Germ-free status was verified through collection of composite mouse and isolator environmental samples, which were subjected to aerobic, anaerobic, and fungal culture. Gram-stained smears of these samples were also examined visually. Isolators were tested weekly during the initial validation and twice monthly once established. The mice used in this study were confirmed free of all bacteria, fungi, and metazoans by these methods.

Viral infection status was verified by quarterly serology for excluded murine pathogens. Mice in the facility are specific pathogen free (SPF) for the following excluded viruses: Sendai virus, pneumonia virus of mice, mouse hepatitis virus, minute virus of mice, Theiler’s murine encephalomyelitis virus, reovirus, lymphocytic choriomeningitis, ectromelia virus, K virus, polyomavirus, mouse adenovirus, rotavirus, mouse cytomegalovirus, hantavirus, mouse parvovirus, and mouse norovirus.

The exclusion list for the SPF facility includes the following bacteria and metazoans, which we know are not present in germ-free mice: Mycoplasma pulmonis, *Helicobacter*, *Pseudomonas*, *Campylobacter*, *Salmonella*, *Citrobacter*, helminth intestinal parasites (pinworms) including *Aspicularis tetraptera* and *Syphacia obvelata*, and ectoparasites (fur mites) including *Radfordia affinis*, *Myobia musculi*, and *Myocoptes musculinus*.

### Preparation of cecal and fecal samples and inoculation of the GF animals.

Cecal contents were collected from 4-week-old conventionally raised Swiss Webster (CONV-D) and C57BL/6 mice following euthanasia with 5% isoflurane until breathing cessation. Confirmation of euthanasia was executed by cervical dislocation and/or decapitation. Mouse cecal contents were expressed from the cecum into a 1.7-ml Eppendorf tube and immediately transferred into the anaerobic chamber. Human fecal samples were collected and stored at −80°C as previously described (40). Surveying identified one as a 35-year-old vegetarian and the other as a 45-year-old omnivore. Samples from the omnivorous donor came from two time points: at year 0 (sample C) and a year later (sample B). Prior to use, cecal and fecal samples were resuspended in 25% (wt/vol) phosphate-buffered saline that was prereduced overnight in an anaerobic chamber. Samples were vortexed for 5 min at 2,500 rpm on a plate shaker, followed by a centrifugation step for 5 min at 200 × *g* to facilitate the removal of the larger particles. For the humanized mouse studies (Fig. 1), 100 µl of the supernatant portion was administered by gavage to each GF mouse in the SW background. All the remaining material was saved and stored in 20% glycerol at −80°C. For the conventionalized mouse studies (Fig. 2), the age, sex, and genetic background of the donors were matched to their recipients (i.e., a GF female SW mouse was colonized with a fecal sample from a WT female SW mouse).

### Gavage and sample collection.

At 4 weeks of age, GF mice were conventionalized with mouse cecal contents or human fecal samples (as indicated in Results) by intragastric gavage. At various times throughout the studies, fecal samples were collected as mice defecated and were stored at −80°C. Calcein (10 mg/kg of body weight intraperitoneally) was injected 7 days and 2 days before euthanasia to evaluate the dynamics of bone formation. Mice were euthanized with 5% isoflurane, continuing for 1 min following breathing cessation. Confirmation of euthanasia was executed by cervical dislocation and/or decapitation. Retro-orbital bleeding was performed for blood collection, and serum separation was achieved by centrifugation at 4°C at 7,500 rpm for 10 min. Serum samples were stored at −80°C. Intestinal sections were collected quickly after euthanasia and stored at −80°C. Cecal contents were expressed from the cecum into a 1.7-ml Eppendorf tube for storage at −80°C. Femurs were fixed in 10% paraformaldehyde for 3 days and then stored in 70% ethanol at 4°C until micro-computed tomography (μCT) imaging. Following imaging, femurs were paraffin embedded for histomorphometry.

### μCT analysis of bone.

μCT was performed as described previously (41). Briefly, femurs stored in 70% ethanol were scanned using a Scanco μCT-40 system (Scanco Medical AG, Brüttsellen, Switzerland) located in the Micro-CT Core at Baylor College of Medicine. Scans were reconstructed at a 16-μm isotropic voxel size. Analysis of trabecular (cancellous) bone was performed by manually contouring a region composed of 75 slices (=1.2 mm) in the distal metaphyseal region of the femur. Parameters including bone volume fraction (BVF; as defined by bone volume per total volume analyzed [BV/TV]), tissue mineral density (TMD), trabecular thickness (Tb. Th.), trabecular number (Tb. N.), and trabecular spacing (Tb. Sp.) were measured using the Scanco software with a threshold value of 250. The length of the femur was measured between the medial condyle and the top of the femoral head. Analysis of cortical bone parameters was executed using an automated thresholding algorithm that is part of the Scanco software package. The region of interest captured a distance spanning 50 slices (=0.8 mm) from the midshaft of the femur toward the distal femur and was measured with a threshold value of 220 using the Scanco software package.

### Static and dynamic histomorphometry.

Fixed femurs (*n* = 4 to 6 per group) were processed as described previously (37, 38). Briefly, fixed femurs were paraffin embedded, sectioned, and analyzed by microscopy. Calcein (100 μl of 20 mg/ml dissolved in sterile saline) was supplemented by gavage at 7 days and 2 days prior to the end of the experiment. Its incorporation into growing bone allowed for bone formation rate (BFR) and mineral apposition rate (MAR) measurements through quantitating the amount of calcein along the bone surface. The distances between the calcein lines and length along the bone surface were used to measure the MAR and BFR, respectively. A commercially available kit (catalog number 387A; Sigma, St. Louis, MO) was used to quantitate tartrate-resistant alkaline phosphatase (TRAP) activity, a measure of osteoclasts present, from sectioned trabecular bone.

### qRT-PCR.

RNA extraction was performed with TRI Reagent (catalog number TR 118; Molecular Research Center, Cincinnati, OH). Synthesis of cDNA was performed with SuperScript III reverse transcriptase (catalog number 8080093; Thermo Fisher Scientific, Waltham, MA). A total of 1 μg of RNA was reverse transcribed. Briefly, an Eppendorf Mastercycler EPS instrument was preheated to 65°C. A mixture containing RNA, 100 ng of random hexamers (catalog number C1181; Promega, Madison, WI), and 1 μl of 10 nM deoxynucleoside triphosphates (dNTPs) (catalog number 18427088; Thermo Fisher Scientific, Waltham, MA) was placed into the thermocycler for 5 min. Following 1 min on ice, a mixture containing the reverse transcriptase and RNaseOUT was added to complete the cDNA synthesis. A cycle of 10 min at 25°C, 50 min at 50°C, and then 85°C for 5 min was used. The cDNA was used immediately or stored at −20°C. The quantitative real-time PCR (qRT-PCR) reaction mixtures contained 1 μl of cDNA, 1 μl each of forward and reverse primer (10 μM), 7 μl of nuclease-free water, and 10 μl of SYBR green PCR master mix (catalog number 170-8882; Bio-Rad, Hercules, CA). A 2-step PCR amplification protocol was used, with acquisition at the annealing and melting curve steps. The protocol included an initial denaturation at 95°C for 30 s, followed by 40 cycles of denaturing at 95°C for 10 s and annealing at 51°C for 20 s. A melting curve was performed at the end at 95°C for 15 s and ramping up from 60°C to 95°C at a rate of +0.2°C/s. Data analysis was performed according to the method described by Pfaffl (42). The primer sequences used are as follows: HPRT forward, 5′ GCTATAAATTCTTTGCTGACCTGCT 3′; HPRT reverse, 5′ AATTACTTTTATGTCCCCTGTTGACTG 3′; TNF-α forward, 5′ AGGCTGCCCCGACTACGT 3′; TNF-α reverse, 5′ GACTTTCTCCTGGTATGAGATAGCAA 3′; IL-1 forward, 5′ TCCCCGTCCCTATCGACAAAC 3′; IL-1 reverse, 5′ GCGGTGATGTGGCATTTTCTG 3′; IL-6 forward, 5′ ATCCAGTTGCCTTCTTGGGACTGA 3′; IL-6 reverse, 5′ TAAGCCTCCGACTTGTGAAGTGGT 3′; 5-HTT forward, 5′ ATTTCCGTTGGTGTTTCAGG 3′; 5-HTT reverse, 5′ CGTCTGTCATCTGCATCCCT 3′; IFN-γ forward, 5′ GGCTGTCCCTGAAAGAAAGC 3′; IFN-γ reverse, 5′ GAGCGAGTTATTTGTCATTCGG 3′; IL-17 forward, 5′ TGAGCTTCCCAGATCACAGA 3′; IL-17 reverse, 5′ TCCAGAAGGCCCTCAGACTA 3′; IL-10 forward, 5′ GGTTGCCAAGCCTTATCGGA 3′; and IL-10 reverse, 5′ ACCTGCTCCACTGCCTTGCT 3′.

### Flow cytometry.

Bone marrow cells were flushed and collected from femurs (*n* = 8 to 10 per group). An amount of 1 × 10^6^ cells was plated per well in a 96-well plate. The staining was performed as described by Collins et al. (43). Briefly, cells were blocked with Fc block (Becton Dickinson Pharmingen, Franklin Lakes, NJ) for 15 min and then stained with antibodies to CD3 (catalog number 56-0032; eBioscience, San Diego, CA), CD4 (catalog number 11-0041; eBioscience, San Diego, CA), CD8a (catalog number 35-0081; eBioscience, San Diego, CA), GR1/Lys-6G (catalog number 53-5931; eBioscience, San Diego, CA), and CD11b (catalog number 12-0113; eBioscience, San Diego, CA) for 30 min. Then, cells were fixed and data were acquired using the Becton Dickinson LSRII flow cytometer. Data analysis was performed using the FlowJo software package (FlowJo, LLC).

### Osteoclast outgrowth assay.

Primary bone marrow cells were isolated from femurs, and amounts of 4.5 × 10^5^ cells per well were plated in a 48-well plate (catalog number CLS3548; Corning, Corning, NY) format. The plated cells were maintained in minimum essential medium alpha (αMEM) (catalog number 12561; Invitrogen, San Diego, CA) plus 10% fetal bovine serum (FBS) (catalog number 16000044; Thermo Fisher Scientific, Waltham, MA) containing 1% penicillin-streptomycin (catalog number 15140122; Invitrogen, San Diego, CA). To induce osteoclast differentiation, cells were stimulated with 30 ng/ml of RANKL (catalog number Q3TWY5; R&D Systems, Minneapolis, MN) and 2 ng/ml of M-CSF (catalog number Q3U4F9; R&D Systems, Minneapolis, MN). The complete growth medium was replenished every 2 days. After 4 days, the cells were fixed and stained for tartrate-resistant acid phosphatase (TRAP) according to the manufacturer’s protocol (catalog number 387A; Sigma-Aldrich, St. Louis, MO). Cells staining for TRAP and multinucleated (>3) were considered osteoclasts.

### DNA extraction from mouse fecal samples, 16S rRNA gene amplification, and sequencing.

For 16S rRNA gene sequencing, DNA was extracted by bead beating followed by the use of the Qiagen DNEasy tissue kit as described previously (37). PCR amplification of the V4 region of the 16S rRNA gene was performed with Phusion high-fidelity DNA polymerase (catalog number M0530; New England Biolabs, Ipswich, MA) using previously described primers and a previously described protocol (40, 44). PCRs were performed in duplicate. Each reaction mixture was composed of 4 μl of template, 1× Phusion high-fidelity buffer, 200 μM dNTPs (catalog number M0530; New England Biolabs, Ipswich, MA), 10 nM forward and reverse primers, 0.2 units of Phusion DNA polymerase, and PCR-grade water to adjust the final volume to 20 μl. A 3-step PCR amplification cycle was used and included an initial denaturation at 98°C for 30 s, followed by 30 cycles of denaturing at 98°C for 10 s, annealing at 51°C for 20 s, and extension at 72°C for 1 min. Replicates were pooled and cleaned using the Agencourt AMPure XP kit (catalog number A63880; Beckman Coulter, Inc., Indianapolis, IN). DNA sample concentrations were measured using the Quant-iT kit (catalog number Q33120; Thermo Fisher Scientific, Waltham, MA) and pooled at equimolar ratios. Sequencing was performed on an Illumina MiSeq platform (Illumina, Inc., San Diego, CA).

### Microbial community analysis.

The MiSeq pipeline in mothur was used to process sequence data (45). The MiSeq pipeline for mothur (essentially as described previously [46] and the MiSEQ standard operating procedure [SOP] version 28 March 2013 [http://www.mothur.org/wiki/MiSeq_SOP]) were used to process sequence data. Following alignment of forward and reverse reads, sequences were quality trimmed and aligned to the Silva 16S rRNA gene reference database formatted for mothur. Sequences were then trimmed to overlap the same region of the 16S rRNA gene and preclustered to clusters with ≥99% identity, and potentially chimeric sequences were identified and removed using the mothur implementation of UCHIME. Sequences were classified according to the mothur-formatted ribosomal database project version 9 (August 2013) using the Bayesian classifier in mothur, and those sequences classified as *Eukarya*, *Archaea*, chloroplast, mitochondria, or unknown were removed. The sequence data were then filtered to remove any sequences present only once in the data set. After building a distance matrix from the remaining sequences with the default parameters in mothur, sequences were clustered into operational taxonomic units (OTUs) with ≥97% similarity using the average-neighbor algorithm in mothur. The taxonomic assignments for each OTU are the majority consensus taxonomic assignment for each sequence within the OTU. Prior to analysis with the phyloseq package of R, additional filtering of the OTU table was done to remove rare OTUs. Namely, those OTUs present in less than 3 samples and/or that contained less than 25 sequences were removed. These filtering steps reduced the number of OTUs from 6,306 to 923 but only decreased the number of sequences per sample by a mean and standard error of the mean (SEM) of 0.7% ± 0.4% (range, 0.1 to 2.3%). The data set included 4,373,773 high-quality sequences. Following the removal of singletons and clustering based off of 97% similarity, 471 OTUs were identified across all samples (*n* = 52), with an average rarefaction depth of 14,920 reads per sample. The cutoff value for inclusion in downstream analyses was representing at least 0.01% abundance for that particular community. Analysis and visualization of microbiome communities were conducted in R, utilizing the phyloseq package to import sample data and calculate alpha and beta diversity metrics (47).

### Statistical analyses.

The results are presented as mean values ± standard errors of the means (SEM). An unpaired *t* test was used to assess differences between groups. The cutoff for significance was a *P* value of ≤0.05. One-way analysis of variance (ANOVA) was applied when more than 2 groups were being compared. For example, a one-way ANOVA was applied between subjects to compare the effect of different microbiotas on bone volume fraction. For microbial community analysis, the levels of significance of categorical variables were determined using the nonparametric Mann-Whitney test for two-category comparisons or the Kruskal-Wallis test for three or more categories. Principal-coordinate analysis (PCoA) plots employed the Monte Carlo permutation test to estimate *P* values (48). All *P* values were adjusted for multiple comparisons by taking into account the false discovery rate (49).
