# Supplementary material for: Microbiota Reconstitution Does Not Cause Bone Loss in Germ-Free Mice
Source: mSphere. 2018 Jan 3;3(1):e00545-17. doi: 10.1128/mSphereDirect.00545-17 (PMC5750390; doi:10.1128/mSphereDirect.00545-17)
Supplement: TABLE S1 [file sph001182436st1.pdf]

Table S1

Condition	B (mm)	b (mm)	D (mm)	d (mm)	t (mm)	C (mm)	c (mm)	I
GF	1.75	0.74	1.06	0.71	0.289	1.31	0.655	0.089151769
GF	1.65	0.9	1.12	0.83	0.317	1.25	0.625	0.088372622
GF	1.68	0.88	1.2	0.82	0.31	1.21	0.605	0.118473972
GF	1.66	0.72	1.2	0.52	0.294	1.54	0.77	0.13559487
GF	1.6	0.85	1.28	0.56	0.297	1.43	0.715	0.15710231
GF	1.64	0.76	1.12	0.75	0.309	1.38	0.69	0.097189389
GF	1.4	0.83	1.12	0.56	0.315	1.23	0.615	0.089235758
A	1.6	0.89	1.12	0.77	0.311	1.36	0.68	0.090236951
A	1.64	0.95	1.2	0.81	0.317	1.41	0.705	0.114123501
A	1.44	0.83	1.1	0.68	0.296	1.28	0.64	0.081127411
A	1.6	0.98	1.2	0.78	0.301	1.42	0.71	0.112687213
A	1.54	0.81	1.12	0.59	0.271	1.36	0.68	0.097864374
A	1.44	0.73	1.12	0.6	0.29	1.36	0.68	0.0914054
B	1.65	0.98	1.12	0.83	0.259	1.5	0.75	0.086131217
B	1.64	0.88	1.2	0.59	0.256	1.36	0.68	0.130006138
B	1.56	0.74	1.2	0.68	0.285	1.48	0.74	0.120687016
B	1.5	0.69	1.12	0.65	0.303	1.42	0.71	0.093977137
B	1.6	0.81	1.2	0.83	0.273	1.3	0.65	0.112780974
B	1.58	0.91	1.12	0.73	0.297	1.24	0.62	0.091423258
B	1.64	0.97	1.2	0.88	0.305	1.46	0.73	0.106471716
C	1.4	0.89	1.12	0.72	0.307	1.22	0.61	0.080100716
C	1.44	0.89	1.2	0.81	0.298	1.2	0.6	0.098751538
C	1.64	0.91	1.2	0.79	0.305	1.42	0.71	0.116877471
CONV-D	1.44	0.87	1.16	0.83	0.29	1.38	0.69	0.085761542
CONV-D	1.44	0.91	1.08	0.71	0.297	1.28	0.64	0.072926027
CONV-D	1.6	0.89	1.08	0.71	0.27	1.36	0.68	0.083152922
CONV-D	1.65	0.83	1.12	0.69	0.272	1.56	0.78	0.100227968
