# Supplementary material for: Microbiota Reconstitution Does Not Cause Bone Loss in Germ-Free Mice
Source: mSphere. 2018 Jan 3;3(1):e00545-17. doi: 10.1128/mSphereDirect.00545-17 (PMC5750390; doi:10.1128/mSphereDirect.00545-17)
Supplement: TABLE S2 [file sph001182436st2.pdf]

**Table S2**

| Condition | B (mm) | b (mm) | D (mm) | d (mm) | t (mm) | C (mm) | c (mm) | I |
|---|---|---|---|---|---|---|---|---|
| GF | 1.16 | 0.8 | 0.88 | 0.56 | 0.165 | 1.06 | 0.53 | 0.031850721 |
| GF | 1.2 | 0.71 | 0.84 | 0.52 | 0.17 | 1.01 | 0.505 | 0.029959243 |
| GF | 1.12 | 0.69 | 0.88 | 0.49 | 0.172 | 1.05 | 0.525 | 0.033421471 |
| GF | 1.16 | 0.77 | 0.84 | 0.51 | 0.156 | 1.11 | 0.555 | 0.028684373 |
| GF | 1.2 | 0.79 | 0.84 | 0.43 | 0.168 | 1.18 | 0.59 | 0.031773279 |
| GF | 1.2 | 0.81 | 0.84 | 0.47 | 0.16 | 1.01 | 0.505 | 0.03073026 |
| GF | 1.12 | 0.71 | 0.88 | 0.5 | 0.169 | 1.13 | 0.565 | 0.033050433 |
| GF | 1.16 | 0.72 | 0.84 | 0.55 | 0.169 | 1.04 | 0.52 | 0.027819585 |
| GF | 1.2 | 0.75 | 0.84 | 0.57 | 0.173 | 1.14 | 0.57 | 0.028045152 |
| GF | 1.12 | 0.76 | 0.88 | 0.45 | 0.168 | 0.99 | 0.495 | 0.034005688 |
| CONV-D | 1.16 | 0.7 | 0.84 | 0.6 | 0.168 | 1.12 | 0.56 | 0.026280495 |
| CONV-D | 1.12 | 0.67 | 0.84 | 0.53 | 0.166 | 1.09 | 0.545 | 0.027639964 |
| CONV-D | 1.08 | 0.69 | 0.92 | 0.51 | 0.165 | 1.15 | 0.575 | 0.036723239 |
| CONV-D | 1.2 | 0.78 | 0.8 | 0.48 | 0.166 | 1.33 | 0.665 | 0.025878774 |
| CONV-D | 1.08 | 0.73 | 0.84 | 0.49 | 0.176 | 0.92 | 0.46 | 0.027157591 |
| CONV-D | 1.08 | 0.75 | 0.8 | 0.44 | 0.149 | 0.93 | 0.465 | 0.023964528 |
| CONV-D | 1.08 | 0.69 | 0.88 | 0.56 | 0.165 | 1.09 | 0.545 | 0.030125921 |
| CONV-D | 1.12 | 0.66 | 0.84 | 0.55 | 0.154 | 1.13 | 0.565 | 0.027147028 |
| CONV-D | 1.04 | 0.73 | 0.92 | 0.49 | 0.166 | 0.92 | 0.46 | 0.035473636 |
