# Supplementary figures and images for: Microbiota Reconstitution Does Not Cause Bone Loss in Germ-Free Mice
Source: mSphere. 2018 Jan 3;3(1):e00545-17. doi: 10.1128/mSphereDirect.00545-17 (PMC5750390; doi:10.1128/mSphereDirect.00545-17)

GF

CONV-D

a)

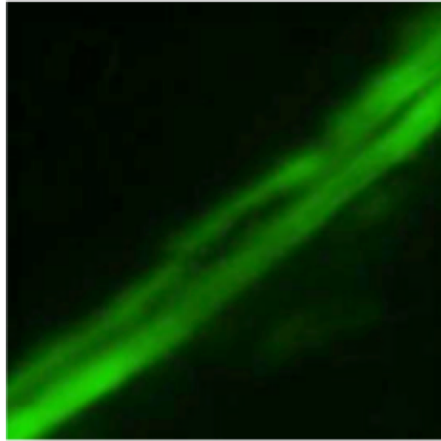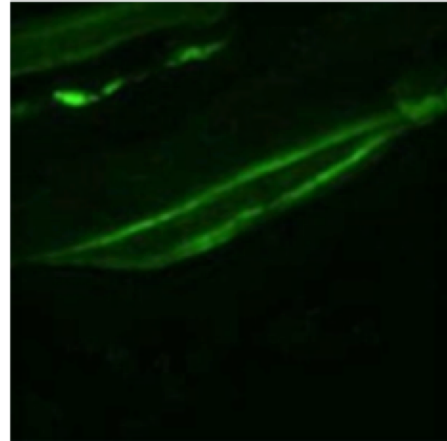

b)

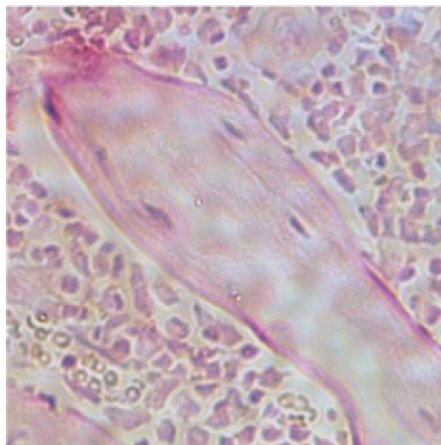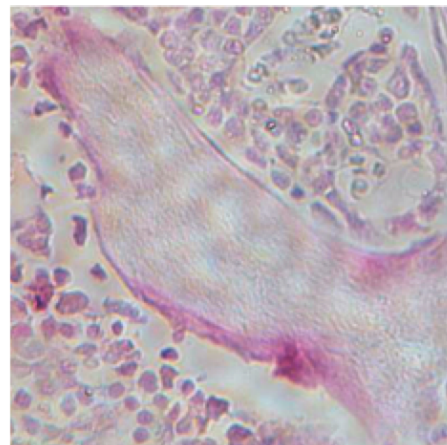

Supplement: FIG S1 [file sph001182436sf1.pdf]

## C57BL/6 Osteoclastogenesis

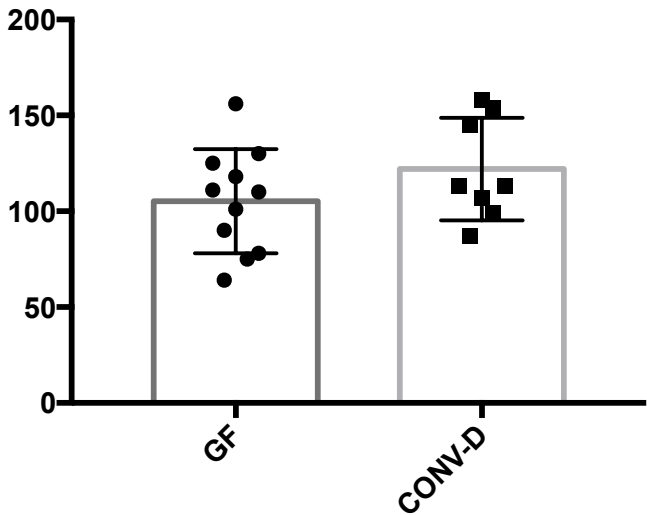

Supplement: FIG S2 [file sph001182436sf2.pdf]
